# A Dynamic and Recursive Process of Resilience in Chinese Families of Children with Autism Spectrum Disorder: A Qualitative Study

**DOI:** 10.3390/bs16101749

**Published:** 2026-09-24

**Authors:** Sijia Guo, Fangru Liu, Sijia Yang, Rujing Huo

**Affiliations:** 1 School of Economics and Management, Dalian Institute of Science and Technology, Dalian 116052, China; sijiaguo@dlmu.edu.cn; 2 College of Public Administration and Humanities, Dalian Maritime University, Dalian 116026, China; ysngsijia@dlmu.edu.cn; 3 Personnel Department, Shandong Open University, Jinan 250014, China; liufangru@sdou.edu.cn

**Keywords:** autism spectrum disorder, caregiving, family resilience

## Abstract

Objective: This study examines how Chinese families of children with autism spectrum disorder (ASD) developed family resilience across their caregiving experiences and identifies the conditions that shape these processes. Methods: In-depth retrospective interviews were conducted with 15 parents and 4 staff members of an ASD service organization in Dalian, China. Reflexive Thematic Analysis was used, with Walsh’s family resilience framework serving as a sensitizing lens. Staff perspectives were treated as supplementary contextual accounts. Results: Three recurring patterns were identified across families, including crisis-oriented disruption and uncertainty after diagnosis, active exploration and family reconstruction during intensive caregiving, and relative stabilization and expanded adaptation as routines and supports settled. These patterns were revisited and combined in different orders rather than following a fixed linear sequence. Resilience was shaped by a mix of intrafamilial and broader contextual conditions. Intrafamilial conditions included intergenerational caregiving and marital relationship. Contextual conditions included cultural norms, stigma, service access, and social support. Parental education was associated with adaptation but was not independent of income and service access. Conclusions: Family resilience in Chinese families of children with ASD should be understood as a dynamic, recursive, and contextually embedded process. The findings point to the need for family-centered, stigma-sensitive interventions that support the wider family and respond to families’ shifting needs over time.

## 1. Introduction

Autism spectrum disorder (ASD) is a neurodevelopmental condition characterized by persistent difficulties in social communication and social interaction, together with restricted and repetitive patterns of behaviors, interests, or activities (Lord et al., 2020). These characteristics vary considerably across individuals in their presentation, severity, and support needs. For many families, particularly when children require substantial support in communication, daily living, behavioral regulation, education, or social participation, caregiving may extend across multiple developmental stages and involve considerable emotional, relational, financial, and organizational demands. In China, due to the influence of Chinese traditional culture, families serve as the primary and core system in terms of providing the care for children with ASD (Song et al., 2013). From this perspective, parents or grandparents of children with ASD normally take the caregiving responsibilities in daily life, and most of them experience stress (Bonis, 2016; Costa et al., 2017), negative emotions (i.e., anxiety) (Lai et al., 2015), caregiving burden (Bozkurt et al., 2019; Marsack-Topolewski & Church, 2019), and even social stigma (Chan & Lam, 2018; Salleh et al., 2020). Furthermore, raising a child with ASD is also a crisis for the family, as it generates a range of challenges and adversities for family relationships and family functioning (Rao & Beidel, 2009; Walton, 2019). Families with ASD children have a similar amount of leisure involvement as families of typically developing children, but reported poorer family functioning, and less satisfaction with family life (Walton, 2019). For instance, externalizing behavior problems often seen in children with ASD often require caregivers to be “on alert”. The need to devote energy and time toward preventing such behaviors may increase anxiety and feelings of exhaustion, which can impair family functioning at a healthy level (Jellett et al., 2015). However, there are several protective factors for families to cope with crisis and adversity, and family resilience is one of them.

Family resilience was initially regarded as a characteristic of family (Bonanno & Diminich, 2013; McCubbin & McCubbin, 1988). McCubbin and McCubbin (1988) define it as the “characteristics, dimensions and properties of families which help families to be resistant to disruption in the face of change and adaptive in the face of crisis situations”. But there had been a disagreement between whether resilience is a process that leads to good outcomes or itself is a good outcome. To respond to this problem, Walsh (2003) defined family resilience as a dynamic process of perseverance, self-governance, and growth, which can deal with crises and challenges. The evolution of the definition highlights the critical role of dynamic process when families navigate adversity. Although there is no consensus about definitions of family resilience, it normally consists of three elements when discussing it, which are the risk, proactive factors, and the outcomes. The present study drew primarily on Walsh’s family resilience framework as its specific emphasis on relational and systematic processes rather than on resilience as an individual trait or a fixed family attribute. Walsh conceptualizes family resilience through three interrelated domains, including belief systems, organizational processes, and communication/problem-solving processes, which are particularly relevant to families living with a long-term developmental condition, such as ASD caregiving. Meanwhile, this framework also emphasizes adaptation through changing adversity, making it suitable for examining how families reorganize meanings, roles, relationships, and resource use over time.

In terms of family resilience of families raising children with ASD, empirical studies have generated an important body of evidence; however, several limitations remain. First, much of the literature has focused on caregivers from an individual-centered perspective rather than a family-centered perspective. Studies have commonly examined parental stress, depression, coping, or individual psychological resilience, and have demonstrated associations between the protective factors related to resilience and caregivers’ well-being (Bitsika et al., 2013; Ruiz-Robledillo et al., 2014). Although these findings are valuable, caregiver adaptation cannot be regarded as family resilience, which involves patterns of shared meaning-making, relational organization, communication, and resource mobilization across family members. Second, existing studies have examined the predictors of resilience, such as social support, acceptance, coping styles, and so on (Luong et al., 2009; McAuliffe et al., 2017; Peer & Hillman, 2014). Such studies identify factors associated with adaptation but provide less insight into how these resources are mobilized, negotiated, and transformed within the family system. Third, although family resilience has increasingly been conceptualized as a dynamic process, empirical studies on ASD families normally operationalize family resilience as a relatively static characteristics measured at one or a limited number of time points. In other words, there is a conceptual gap between the theoretical perspective of family resilience as an ongoing process and empirical approaches that primarily assess family resilience as an outcome. Therefore, qualitative research is particularly valuable for examining how families retrospectively interpret changes in their beliefs, relationships, caregiving arrangements, and engagement with social resources across different periods of their caregiving experience.

Besides, the Chinese context provides a particularly relevant setting in which to examine family resilience. Families remain central providers of long-term care and support for children with developmental disabilities, while parents and grandparents often share responsibilities for daily caregiving, rehabilitation, education, and social participation. At the same time, families may encounter uneven access to specialized services, substantial financial and time demands associated with rehabilitation, and stigma surrounding developmental disability. Intergenerational caregiving can provide important practical and emotional resources, but differences in beliefs about diagnosis, treatment expectations, and parenting may also generate family tension. These sociocultural and service-system conditions suggest that family resilience is shaped not only by intrafamilial processes but also by families’ interactions with broader social, cultural, and institutional environments.

Building on these gaps in the literature, the present study conceptualized family resilience as an ongoing and contextually embedded process of adaptation rather than as a fixed trait or a predetermined developmental sequence. We used retrospective qualitative interviews to examine how parents of children with ASD made sense of changes in family beliefs, organizational arrangements, communication, and resource use across their caregiving experiences. Interviews with staff from an ASD service organization were included as supplementary contextual accounts to illuminate recurring patterns observed across families and the broader service environment.

This study addresses the following research questions:

RQ1: How do parents retrospectively describe changes in family beliefs, organizational processes, and communication while adapting to raising a child with ASD?

RQ2: What recurring patterns and turning experiences characterize families’ accounts of resilience adaptation?

RQ3: What individual, familial, and social–contextual conditions appear to facilitate or constrain these family resilience processes?

Rather than seeking to establish a universal developmental trajectory, this study aimed to generate an interpretive account of family resilience processes as retrospectively described by participants and to identify implications for family-centered social work and psychosocial intervention.

## 2. Materials and Method

### 2.1. Participants

The present study adopted the concept of information power to determine the sample size. According to this principle, the required number of participants depends on the aim, specificity, and depth of the interview. The focused aim and the depth of the interviews led us to stop recruiting once the data reached sufficient richness and relevance.

Specifically, a single interviewer carried out all the interviews, and the interview phase ceased when the collected data were assessed to meet the standards of sufficient richness and relevance. The participants consisted of 15 parents of children with ASD and 4 staff members from Agency A, all Chinese nationals. To illustrate the heterogeneity among families, participants were differentiated in terms of family income and educational background, including six from high-income families, six from middle-income families, and three from low-income families. The total number of college-educated participants was eight (see Table 1 and Table 2).

### 2.2. Researchers

The data collection and analysis team consisted of three members, all Chinese and all trained in social work and psychology. The lead researcher, R1, a female master’s student concentrating on autism and family resilience study, was responsible for the interview conduction, data transcription, and primary data analysis. R2, a female master’s student with the same research focus, contributed to discussions on the interview protocol and coding results. Additionally, R3, a professor with extensive practical and research experience in the field of children with autism spectrum disorders and family resilience, acted as a critical friend, providing ongoing methodological guidance to R1 and prompting critical reflection on the developing analysis.

The research team had a strong background in the field of family resilience, children with ASD, and qualitative research. This background allowed this study’s focuses on the difficulties and the reality of families with ASD children from a specialized and sensitive perspective to be assessed. However, the study may also introduce potential biases, such as selective attention or overinterpretation. To address such limitations, the team deliberately engaged in long-term, immersive contact with families with ASD children during this study to gain in-depth insights into their real life and core needs. Throughout the entire research process, we continuously reflected on our professional backgrounds, positional biases, and assumptions. We strove to ensure that interview questions aligned with the actual experiences of families with ASD children.

### 2.3. Data Collection

#### 2.3.1. Participant Recruitment

Ethical approval has been granted by Dalian Maritime University (No. 172401), prior to data collection. This study recruited participants using purposive sampling. Families with ASD children were included in this study if they met the following eligibility criteria: (a) one or more of their children has been diagnosed with ASD by a specialized physician, (b) they must be the primary caregiver of a child with ASD, or (c) service providers who have at least 1 year of work experience and are willing to share their experience and feelings. Thus, this study has a diverse sample to understand the family resilience of families with ASD children.

The recruitment announcement for this study was disseminated by researcher R2, who had access to Agency A. Agency A is a professional social organization in China that provides social integration, capacity enhancement, and rehabilitation training services to ASD children and their families. Recruitment combined purposive sampling and snowball sampling methods. Parents and staff members who were interested in this study could register with the head of Agency A. Then, R1 contacted eligible participants and provided details about this study, including the study’s aims, procedures, ethical considerations, audio recordings, and participants’ right to withdraw from this study at any time without providing a reason. Verbal informed consent was obtained from the participants for two main reasons. First, written consent might place pressure on parents of children with ASD who already bear the stigma. Second, the interviews were initiated face to face, and the study’s aims, procedures, and the right to withdraw were fully explained during recruitment.

#### 2.3.2. Interviews and Transcription

R1 drafted the interview protocol and sought revision suggestions from R3. Meanwhile, interview protocols were completed by professionals who provided services for ASD, and physicians were consulted. In addition, we also designed an interview protocol for the staff of Agency A. This allowed us to describe the whole process of resilience for the families with ASD children in a more comprehensive and multidimensional way.

The interview focused on the changes that occurred in the family, their attitudes toward ASD, and the way family members communicate and interact. The research purpose was translated into concrete interview questions that resonated with participants’ lived experiences to elicit authentic and in-depth responses.

Each interview was conducted in a closed, undisturbed space to minimize the influence of others’ presence and foster an atmosphere of trust, understanding, and openness. With participants’ consent reaffirmed before each session, all interviews were audio-recorded. Upon completion, participants were thanked.

All interviews were conducted in Mandarin Chinese, and data coding and theme development were carried out on the Chinese transcripts to preserve the nuance of participants’ accounts. Quotations selected for presentation in this manuscript were translated into English by R1, a bilingual researcher with native proficiency in Chinese and fluency in academic English. Translation is recognized as an interpretive act rather than a neutral process, shaping the data available for analysis (Squires, 2009; Temple & Young, 2004). We therefore prioritized meaning equivalence over literal rendering, consistent with recommendations for cross-language qualitative research (Van Nes et al., 2010). Translation choices were discussed within the research team, particularly where expressions carried culturally specific meanings that risked being flattened or distorted in English. This study also used a denaturalized transcription approach (Point & Baruch, 2023). In this process, repetitive words were removed, sentence structures were refined, and punctuation was standardized to improve clarity while preserving participants’ intended meanings. Verbal fillers and natural speech styles were retained. Finally, the researcher ensured that these procedures were consistent with the original meaning, tone, or emphasis.

To follow ethical rules for data handling, all audio files were renamed with number codes before being uploaded for transcription, and the transcribed texts used the same numbering system. Also, any personal information that could identify someone (like names, places) in the transcripts was made anonymous.

The total recording duration was 1271 min, with individual interviews ranging from 42 to 104 min (M = 66.9, SD = 15.9). The transcripts totaled 285,992.00 Chinese characters, with individual transcripts ranging from 10,727 to 20,518 Chinese characters (M = 15,052.3, SD = 3585.0).

### 2.4. Data Analysis

The purpose of this study was to explore the dynamic processes of the generation of family resilience in families with ASD children and to identify core themes, rather than to achieve coding objectivity. Accordingly, the data were analyzed using Reflexive Thematic Analysis (Braun & Clarke, 2021a). This approach conceptualizes researcher subjectivity as a valuable resource for knowledge generation, rather than a threat that must be controlled. It emphasizes an open, flexible, and iterative coding process instead of relying on a predetermined coding framework. In addition, coding quality in Reflexive Thematic Analysis is evaluated through theoretical sensitivity, reflexivity, and interpretive depth, rather than through coder consensus or inter-rater reliability.

We collected interview data from both parents and staff members. Staff perspectives were treated as supplementary to the parents’ accounts, as they offered an outside view of family functioning and service use.

All interview data were managed and analyzed using NVivo 12, a qualitative data analysis software that supports thematic analysis. The coding process broadly followed the six-step framework for Reflexive Thematic Analysis (Braun & Clarke, 2021b), with R3 acting as a critical friend. Data analysis was primarily inductive and data-driven. The analysis proceeded as follows.

1. During transcript proofreading, R1 revisited the data to become familiar with the interviews and to consolidate initial impressions (initial impressions were formed during the interviews).
2. R1 read all transcripts, drafted preliminary subthemes, and reflected on personal assumptions and potential biases. She remained attentive to how her personal stance shaped her reading of the data and treated low engagement in reflection as a phenomenon to explore. Reflection on assumptions and biases continued throughout the analysis.
3. Then, R1 proceeded the formal analysis, generating and iteratively refining codes. During this process, R3 provided ongoing methodological guidance based on extensive research experience. Finally, R1 established a clear and systematic coding framework.
4. R1 and R2 discussed the coding framework, and R1 revised the coding. R3, acting as a critical friend, reviewed the developing analysis and challenged R1’s interpretations through supervisory dialog. When disagreements arose, the team returned to the transcripts together and refined the themes against the original data.
5. When disagreements arose, the team returned to the transcripts together and compared the different interpretations against the original data and determined more appropriate themes. After reviewing the feedback, R1 and R2 finalized the analysis results.

Drawing on Braun and Clarke’s (2022) description of good Reflexive Thematic Analysis, this study adopted the following measures to ensure credibility:

1. R1 continuously reflected on her own stance during both the interview and analysis stages.
2. As the primary coder, R1 had extensive experience in qualitative research, as did R2 and R3.
3. R2 and R3 worked as critical discussion partners, which helped broaden and enrich the analytical perspective.
4. During the analysis, R1 deeply immersed herself in the data. And revisions were developed through team discussion.
5. The research team has maintained a lengthy focus on children with autism spectrum disorder and family resilience, which helped ensure that data interpretation closely aligned with real-world contexts.
6. The coding results were reviewed by a staff member with rich experience, who largely endorsed them.

## 3. Findings

Based on in-depth interviews with 15 parents and 4 staff members of children with ASD, this study found that there are three recurring patterns of family resilience adaptation, including crisis-oriented disruption and uncertainty, active exploration and family reconstruction, and relative stabilization and expanded adaptation. These patterns were primarily developed from parents’ retrospective accounts of how family beliefs, organizational arrangements, communication, and resource use changed across their caregiving experiences. Staff interviews were used as supplementary contextual accounts, particularly to illuminate recurring family difficulties, service engagement, and patterns observed across multiple families.

These three patterns are presented sequentially for analytical clarity; however, they should not be interpreted as universal developmental stages through which all families necessarily progressed in the same order. Participants’ accounts varied in the intensity, duration, and combination of these patterns. Some families described relatively rapid movement from initial uncertainty toward active coping, whereas others continued to experience conflict, anxiety, or uncertainty even after they had developed more stable caregiving arrangements. Moreover, several participants described renewed concerns when confronted with changing demands, such as children’s education, employment, long-term care, or the aging of caregivers. Family resilience was therefore understood as a dynamic and recursive process rather than a fixed or irreversible developmental trajectory.

Across participants’ narratives, several experiences appeared to be associated with meaningful shifts in family adaptation. These included receiving a formal ASD diagnosis, gaining access to professional intervention or support, adjusting family caregiving roles, and gradually shifting attention from immediate crisis management toward longer-term planning and broader social participation. These experiences did not function as standardized transition points for all families, but they helped participants retrospectively organize and interpret changes in their family lives.

### 3.1. Recurring Patterns of Family Resilience Development

#### 3.1.1. Crisis-Oriented Disruption and Uncertainty

The first recurring pattern was characterized by disruption, uncertainty, and difficulties in making sense of the child’s diagnosis. For many parents, receiving an ASD diagnosis represented a major challenge to previously held expectations about the child and family life. Participants described confusion, worry, denial, and disagreements among family members regarding the meaning of the diagnosis and the child’s future. However, the intensity and duration of these responses varied across families.

In terms of family belief systems, some participants described difficulty accepting the diagnosis, particularly when family members held different interpretations of the child’s condition. Expectations that the child would eventually “recover” or become similar to typically developing peers sometimes delayed acceptance of the diagnosis. Staff participant B17 described such reactions as recurring among some families receiving services:

*“In fact, many families think that children are still young. Although they just have not fully developed currently, they can recover in future. They refuse to accept the fact that their child is diagnosed with autism and are unwilling to let the child go outside. Instead, they hide the child, in order to restrict their child’s contact with outside world.”*

(B17)

Differences in diagnostic acceptance were also evident within families. B10 described a contrast between her own acceptance and the attitude of an older family member:

*“My child’s grandmother still can’t accept the reality that our child has been diagnosed with autism. I accepted it relatively quickly. Child’s father and I were both preparing for the worst result and striving for the best. But my child’s grandmother imagines the best while acting the worst.”*

(B10)

These accounts suggest that the diagnosis was not interpreted uniformly within the family. Intergenerational differences, previous beliefs about disability, expectations concerning children’s development, and concerns about social evaluation could all shape how family members understood the diagnosis.

Meanwhile, the diagnosis also disrupted existing organizational arrangements within some families. Participants described the need to renegotiate caregiving responsibilities, employment, household labor, and interactions with extended family members. In some cases, these demands intensified existing family pressures; in others, they prompted family members to mobilize internal resources and assume new responsibilities.

For example, B4 described experiencing multiple concurrent family stressors while her husband began seeking information and external support:

*“Following my mother passing away, our family underwent a series of changes and tensions arising between me and my maternal relatives. At the same time, we discovered our child’s unusual behavior, and I could not bear the pressure at all. My husband then began reading books, attending lectures, and engaging with social organization to help our family navigate this situation.”*

(B4)

In contrast, B8 described conflict between generations regarding how the child’s condition should be understood:

*“When my family still could not accept this, many conflicts arose between the older generation and me at home. They felt my husband and I did not raise this child properly and insisted there was nothing wrong with the child’s health.”*

(B8)

These narratives illustrate that family disruption following diagnosis was not determined solely by the diagnosis itself. Rather, difficulties emerged through the interaction among caregiving demands, existing family relationships, role expectations, and access to explanatory or professional resources.

Additionally, communication was another important aspect of strain. Some participants reported difficulty speaking openly about the diagnosis, especially when family members feared stigma or anticipated negative reactions from relatives. Staff accounts also indicated that diagnostic information was sometimes concealed within families:

*“Some parents have accepted the reality that their child is diagnosed with ASD, but the grandparents have not. So the parents end up taking their child to interventions or treatments without telling the grandparents. Also, there are sometimes differing views between mothers and fathers. For instance, in our agenda, one father secretly engaged his child in interventions and activities.”*

(B17)

B10 similarly described concealing the diagnosis from grandparents because of anticipated distress and social expectations:

*“I still cannot tell my child’s grandparents the truth, as they would not be able to accept this fact. Besides, my child is a boy, and in our village a lot of expectations are placed on him.”*

(B10)

At the same time, this study also found that conflict and concealment were not inevitable. Some families began modifying their communication in response to concerns about the child’s emotional environment. B4 described deliberately reducing marital conflict:

*“At that time, my child’s father and I would often argue over household matters. But later, I realized this atmosphere would negatively affect our child’s development. So we reached an agreement to gradually reduce our arguments and cultivate a family environment more conducive to our child’s growth.”*

(B4)

Thus, the crisis-oriented pattern was characterized not only by distress but also by the beginning of adaptive efforts. Even during periods of uncertainty, some family members had already begun seeking information, reorganizing roles, and modifying communication.

#### 3.1.2. Active Exploration and Family Reconstruction

The second recurring pattern involved a shift toward more active engagement with the child’s condition and the practical demands of caregiving. Participants described learning about ASD, seeking intervention, adjusting expectations, redistributing caregiving roles, and exploring both internal and external resources. This pattern was not necessarily preceded by a prolonged period of denial, nor did it occur at the same point in time across families.

In terms of belief systems, several parents described a gradual movement from uncertainty toward greater acceptance and more pragmatic expectations. For instance, B1 reflected on this change as an incremental process:

*“Later, my mindset gradually shifted, and I slowly began taking my child for medical diagnoses, applying for a disability certificate, and handling the related procedures. The entire process unfolded quite naturally.”*

(B1)

This account illustrates that acceptance did not necessarily occur as a single event. Instead, it could develop through engagement with services, caregiving responsibilities, and repeated encounters with the practical realities of the child’s needs.

Participants also described changes in family organization, particularly in the distribution of caregiving responsibilities. B3 described how her husband gradually became more involved after observing changes in their child:

*“At first, I completely understood my husband’s avoidance of this issue. I proactively spent more time with our child and tried to influence him through my actions. Later, as he noticed the changes in our child and received positive feedback, he gradually took on more responsibility. Now he spends more time with our child than I do.”*

(B3)

This example illustrates how family roles could be renegotiated through ongoing interaction rather than through a single structural adjustment.

A further characteristic of this pattern was the growing use of external resources. Some families joined parent mutual-support groups, engaged with professional organizations, or actively sought information about intervention and educational options. Such engagement appeared to provide not only practical information but also emotional reassurance and a sense of shared experience.

B4 highlighted the emotional importance of peer support:

*“The parental mutual support group provided me with tremendous emotional power. I discovered that my child behaved better when we were in a more positive emotional state. Now, our family life gradually returned to a normal condition with a stronger family bonding, that is my husband and I collaborate with each other to raise this child.”*

(B4)

The value of external support was therefore not limited to service provision. For some parents, contact with other families reduced isolation and helped them reinterpret their own experiences.

Participants also described changes in how they understood intervention and parenting. Early caregiving efforts were sometimes characterized by intensive attempts to acquire professional techniques. Over time, however, some families developed more individualized and relational ways of interacting with their children. B6 described this shift:

*“At the beginning, the whole family rushed to learn intervention methods, rehabilitation approaches, and ABA techniques, which made everyone quite tense because these were professional methods. Later, however, we found that simply lying on the bed with the child and chatting, while we did our own thing and he listened to his music. Without intervening, just being present and accompanying him, actually worked quite well.”*

(B6)

This reflects a broader change in family adaptation, where parents were not simply acquiring more intervention knowledge but were also revising expectations about what constituted effective caregiving and meaningful interaction. In this sense, active reconstruction involved both practical problem-solving and changes in family meaning-making.

#### 3.1.3. Relative Stabilization and Expanded Adaptation

The third recurring pattern involved relative stabilization, characterized by more established caregiving routines, clearer role arrangements, greater acceptance of the child, and increased ability to mobilize external support. During this period, participants also continued to describe uncertainty, worry, or unmet needs; therefore, this period is “relatively” stable, and stabilization did not indicate the absence of negative emotions.

Within this period, regarding family belief systems, several parents described a shift away from expectations of a “cure” toward greater acceptance of the child’s characteristics and a more realistic understanding of long-term caregiving. B3 described a more positive reinterpretation of family life:

*“Now we feel that the child isn’t bad at all. He can always stay with us. He doesn’t need to find a wife or get married, and we don’t have to help take care of grandchildren. There’s no academic pressure. In that sense, it’s actually quite good. So our mindset has gradually become better and better.”*

(B3)

B7 similarly described becoming more accepting and tolerant:

*“I’m actually very introverted, but after having this child, I’ve become especially accepting of him… I’ve become very tolerant, able to accept many things… So I feel that I’m happier.”*

(B7)

These accounts suggest that adaptation sometimes involved changes in the meaning attributed to caregiving and family life, rather than simply reductions in stress.

Notably, greater acceptance did not eliminate negative emotions, as some parents who described relatively stable family functioning continued to worry about adulthood, employment, independent living, and long-term care.

For instance, B4 stated:

“Anyway, I feel that my mindset is still fairly good. I’ve been staying at home taking care of her for more than twenty years. But of course, there is still anxiety and worry, because there are definitely concerns about the future.”

(B4)

B6 similarly focused on employment:

“She graduated from a vocational secondary school. I just hope she can have a regular, formal job. Ideally, she could take a short rest at noon and then work well in the afternoon.”

(B6)

Organizationally, some participants described relatively stable caregiving divisions. Mothers often remained heavily involved in everyday care, while fathers or grandparents assumed complementary roles. For example:

“Her father is mainly responsible for earning income and supporting the family financially, while I take primary responsibility for taking care of our child.”

(B7)

Another participant described a shared caregiving arrangement involving a grandparent:

“During the day, her grandmother takes care of her. After work, I take over. I am not a full-time caregiver. I participate in the treatment in the morning and I also take the child to kindergarten in the afternoon.”

(B9)

Furthermore, this period was also associated with a greater orientation toward community inclusion, future planning, and social support. Some parents moved beyond immediate rehabilitation concerns and began considering supported employment, community living, long-term care, or broader social acceptance. B9 stated:

“So now we also feel that the next step is to explore supportive employment, like independent community living. As parents grow older and may no longer be able to care for the child, we are thinking about whether there will be an alternative social service system.”

(B9)

B8 similarly emphasized inclusion:

“This is probably a lifelong intervention process. Although we work very hard to take care and do interventions, I feel the results often do not meet expectations. Now we can only hope that society will be more inclusive and accepting of them. Greater understanding and tolerance would be enough. Allowing them to integrate into the community is the direction of my efforts.”

(B8)

These suggest that family resilience increasingly extended beyond intrafamilial adjustment toward the family’s relationship with broader educational, service, community, and policy environments.

### 3.2. Contextual and Family Conditions Associated with Family Resilience

In line with the interviews, educational background is associated with how families accessed, interpreted, and used ASD-related information. In other words, participants with more educational or professional resources sometimes described early information seeking, comparison of intervention options, and more active engagement with professional services. For example, B7 described a relatively rapid move toward information seeking and problem-solving rather than prolonged denial. However, the findings of this study did not support the educational level of caregivers as an independent determinant of family resilience. Education was intertwined with other important factors, such as family income, occupation, social resources, and so on. Therefore, educational background is better regarded as one important component of resource-navigation capacity rather than a direct causal predictor of family resilience.

In addition, this study also highlighted the importance of family members who assumed central roles in organizing caregiving, seeking information, and coordinating family responses. The importance of these central family members did not mean that resilience was an individual characteristic transferred to the family. Rather, their influences operated through family relationships. Specifically, they mobilized resources, shaped interpretations, negotiated caregiving responsibilities, and sometimes influenced the engagement of other family members. B3’s account of her husband gradually becoming more involved provides one example of this relational process.

At the same time, heavy reliance on one family member could also create vulnerability. When caregiving coordination depends disproportionately on one person, particularly the mother, emotional exhaustion or reduced access to support may affect the functioning of the wider family system.

Moreover, social support was evident across all three patterns of family resilience development. Participants referred to professional organizations, parent groups, schools, relatives, and community resources as sources of emotional, informational, and practical assistance. Therefore, access to social support influences the process of family resilience development. First, professional information helped families have a better understanding about ASD and make rational decisions about intervention. Second, peer contact reduced social isolation and enhanced emotional support. Third, practical support from external resources, such as relatives or professional institutions, could decrease caregiving burden through satisfying needs.

## 4. Discussion

In line with the findings of this study, there are several important aspects that are worth being discussed in further detail.

### 4.1. Family Resilience as a Dynamic and Recursive Adaptation Process

The central finding of this study is that family resilience in the context of ASD, as described by primary caregivers, was not a fixed family characteristic or a uniform developmental trajectory, but an ongoing and recursive process of adaptation. Participants’ retrospective narratives reflected three recurring configurations, which are crisis-oriented disruption and uncertainty, active exploration and family reconstruction, and relative stabilization and expanded adaptation. These configurations were analytically distinguishable but not mutually exclusive, irreversible, or universally sequential.

Meanwhile, this interpretation is broadly consistent with the conceptualization of family resilience, which emphasizes adaptation, reorganization, and meaning-making in response to changing adversity (Walsh, 2003). In line with the perspective of life-course and chronic illness, families may repeatedly reorganize when confronted with new developmental or contextual demands (Rolland, 1987). In ASD caregiving, the families often face diverse challenges after diagnosis as well, such as educational transitions, behavioral regression, financial pressure, or long-term caregiving (Lounds et al., 2007). Therefore, although the family enters a “stabilization” period, it does not mean that there is no distress or challenges for the family. In other words, family could function adaptively while simultaneously experiencing anxiety, fatigue, or uncertainty.

### 4.2. Contextual Factors Shaping Family Resilience

The findings of this study also indicate that family resilience is shaped by contextual factors. First, the educational background of caregivers appears to be associated with their capabilities of information seeking and service use. Education is closely intertwined with income, occupation, social networks, resource utilization, and adaptive functioning (Meadan et al., 2010; Xue et al., 2014). From this perspective, families with limited educational resources may face structural barriers that limit their access to high-quality information and intervention, whereas families with substantial educational resources may be better positioned to mobilize available support (Schwarzer & Knoll, 2007).

Second, this study also finds that key family members play important coordinating roles. In many families, mothers assumed substantial responsibility for identifying the child’s needs, arranging services, and conducting caregiving. This is consistent with previous studies indicating the central role of mothers in ASD caregiving in China (Chen et al., 2025; Feng et al., 2025). This finding does not mean that mothers themselves generate family resilience. Rather, their influence operates through relational processes, including mobilizing other family members, negotiating roles, and maintaining access to support. Meanwhile, it is worth noting that the concentration of caregiving responsibility on mothers creates potential tension regarding family resilience. This means a highly involved, central caregiver may help organize family adaptation, but may also experience disproportionate emotional and practical burdens. Thus, resilience-oriented intervention should avoid relying on maternal sacrifice as the primary mechanism of family adaptation and resilience.

Third, social support is a critical protective factor across all family resilience developmental stages, particularly during the stabilization phase. Families benefited substantially from emotional support, informational guidance, and practical assistance provided by professional agencies, parental mutual-support groups, educational institutions, and broader community networks (Lei & Kantor, 2021; McCabe, 2008). Consistent with the ecological systems theory, these findings demonstrate that family resilience is not generated solely within the family itself, but rather through continuous interaction between families and their surrounding social environments.

These three elements work together to boost resilience. Their dynamic interaction adds to Walsh’s family resilience framework (Walsh, 2016) by making clear how ASD families build resilience. Importantly, these processes do not operate alone, but coexist and are shaped by the multi-layered ecological context of Chinese society. Effective intervention and support for ASD families therefore require attention to the interplay among microsystem (parental cognition and roles), mesosystem (social capital and community connections), and macrosystem (cultural and policy environment) levels. Resilience is not just a mix of separate factors. Instead, it is the result of their interactions. This idea has important meanings for intervention work. Focusing only on one factor probably will not result in effective improvement. Instead, interventions should cover all three areas. This way, we can truly boost family resilience.

### 4.3. The Sociocultural Embeddedness of Family Resilience in China

The family resilience processes identified in this study were embedded in a particular social and cultural context. Chinese family norms concerning interdependence, intergenerational responsibility, caregiving duties, and social reputation served as both resources and constraints.

On one hand, a strong sense of family responsibility supports continuity in caregiving. Participants frequently described mothers, fathers, and grandparents sharing daily caregiving tasks, indicating that long-term care in these families was organized around the family as a whole. This aligns with prior research showing that Chinese families tend to treat disability-related caregiving as a shared family responsibility rather than a task falling on one parent alone (Holroyd, 2003; Zhao & Fu, 2022).

On the other hand, stigma and concern about social evaluation sometimes make it harder for families to accept the diagnosis and seek help. Some participants concealed the diagnosis from relatives or limited their social participation in anticipation of stigma. These experiences echo earlier work on affiliate stigma among Chinese caregivers of children with ASD (Chan & Lam, 2018; Mak & Kwok, 2010).

Intergenerational caregiving illustrates this duality. Grandparents offer substantial practical support, but disagreements between generations over the diagnosis, treatment expectations, and parenting practices also generate conflict. This means that, in the same family, support and tension can coexist.

These findings also caution us against treating Chinese families as uniformly collectivist and Western families as uniformly individualist. In this study, resilience had to be understood within each family’s own relationships, service setting, and cultural expectations. Cultural norms shape the options families have for adaptation, but families negotiate them in daily life rather than simply following these norms.

### 4.4. Implications for Social Work and Psychosocial Intervention

The findings suggest several implications for social work and psychosocial intervention. Because family resilience changes over time and fluctuates rather than unfolding through fixed stages, intervention should fit the family’s current needs and avoid assuming that every family follows the same trajectory.

Families still uncertain after the diagnosis may benefit from accessible psychoeducation, emotional support, counseling that is sensitive to stigma, and family conversations that help all members understand ASD together. Social workers can also help parents and grandparents work through their differences and reduce blame and secrecy within the family (Guo et al., 2026).

When families are actively rebuilding their caregiving arrangements, intervention can focus on how roles are negotiated, how caregiving is shared, how family members communicate, and how families identify and use services and peer support. Support should not target only the main caregiver; where possible, it should reach the wider family, with particular attention paid to preventing an undue concentration of caregiving responsibilities on mothers.

For families in more stable circumstances, service needs often shift toward longer-term concerns, including school transitions, employment, supported living, the aging of caregivers, future guardianship, and community inclusion. These concerns do not signal a loss of resilience but reflect new developmental and social demands that require further adaptation.

At the community and policy levels, social work can strengthen links among families, rehabilitation organizations, schools, community services, and employment systems. Peer networks are particularly valuable because they offer families both practical knowledge and a sense of belonging. Reducing stigma and improving access to inclusive services are therefore not separate issues; that is, both are part of the broader social conditions that allow families to sustain resilience over time.

### 4.5. Strengths and Limitations

This study has several strengths. It examined the family as a whole rather than focusing on stress experienced by the individual caregiver, and it purposefully included parents varying in income levels, education status, and family configuration. Retrospective narratives were used to examine how families made sense of changes over time, and staff interviews provided a complementary perspective on how families engaged with services and on difficulties that recurred across families.

Several limitations should also be noted. First, the interviews were conducted at a single time point and relied on retrospective accounts. The findings therefore reflect how parents comprehend their experiences in the present to some degree; they probably could not establish a fixed sequence among the patterns or demonstrate that one pattern causes another. Future longitudinal qualitative research could follow families over time as they encounter new transitions.

Second, all participants were recruited through a single social service organization and had already had some contact with formal support. Families who find services difficult to access, have left professional services, or face greater social disadvantage may adapt in different ways.

Third, parent and staff interviews drew on different standpoints. This means parents shared their own family experience, whereas staff offered professional observations across many families. The two data sources were analyzed separately, but they represent different forms of evidence.

Finally, most families were represented by only one caregiver, and the views of the autistic children themselves were not included. Future research that involves multiple family members and the autistic person could offer a fuller account of family resilience as a relational process.

## 5. Conclusions

This study examined how Chinese families of children with ASD generated family resilience across their caregiving experiences. The central finding is that family resilience is not a uniform developmental trajectory. Instead, it is a dynamic and recursive process in which families move between three recurring patterns. These patterns are crisis-oriented disruption and uncertainty, active exploration and family reconstruction, and relative stabilization and expanded adaptation. They appear in different orders and combinations across families.

Family resilience was shaped by both intrafamilial and broader contextual conditions. Within the family, intergenerational caregiving, central family members, and social support all mattered. Outside the family, cultural norms of interdependence and stigma, together with uneven access to professional services, shaped the options available to families.

These findings carry practical implications for social work. Because families move through these patterns at different paces, support should be tailored to the family’s current needs rather than applied according to a fixed-stage model. Services should reach the wider family rather than focusing only on the primary caregiver, and they should be sensitive to the stigma that families face. Reducing stigma and improving access to inclusive community services are themselves part of sustaining family resilience over time.

## Figures and Tables

**Table 1:** Demographic information of caregiver participants.

Participant	Gender	Family Income	Education Level	Child’s Diagnosed Age	Child’s ASD Severity (Self-Report)	Child’s Care Arrangement
B1	Female	High	Master’s degree	2.5	Moderate	Family care + Agency day care
B2	Female	High	Associate degree	6	Mild	Family care + Agency day care
B3	Female	High	Doctorate	3	Moderate	Shadow teacher + Agency day care + Family care
B4	Male	Medium	Associate degree	2.5	Moderate	Family care + Agency day care
B5	Female	Low	High school	7	Mild	Family care + Agency day care
B6	Female	Low	Technical secondary school	3	Moderate	Family care + Agency day care
B7	Female	High	Doctorate	3	Moderate	Shadow teacher + Agency day care + Family care
B8	Female	Medium	Associate degree	2.5	Moderate	Family care + Agency day care
B9	Male	Medium	Undergraduate degree	3	Mild	Primarily family care
B10	Female	Medium	Undergraduate degree	3	Mild	Primarily family care
B11	Female	Medium	High school	3	Severe	Family care + Agency day care
B12	Male	Low	Associate degree	4	Moderate	Primarily family care
B13	Female	High	Undergraduate degree	3.5	Mild	Family care + Agency day care
B14	Female	High	Undergraduate degree	2	Severe	Shadow Teacher + Family care + Agency day care
B15	Male	Medium	Undergraduate degree	2	Moderate	Primarily family care

**Table 2:** Demographic information of staff participants.

Participants	Gender	Age	Position
B16	Female	40	General manager
B17	Female	34	Project manager
B18	Male	24	Staff of publicity department
B19	Female	26	Staff of project department

## Data Availability

The data supporting the findings of this study are available from the corresponding authors upon reasonable request. The data are not publicly available due to to ethical and privacy concerns regarding the sensitive interview data of participating families.
